# The Potent Respiratory System of *Osedax mucofloris* (Siboglinidae, Annelida) - A Prerequisite for the Origin of Bone-Eating *Osedax*?

**DOI:** 10.1371/journal.pone.0035975

**Published:** 2012-04-25

**Authors:** Randi S. Huusgaard, Bent Vismann, Michael Kühl, Martin Macnaugton, Veronica Colmander, Greg W. Rouse, Adrian G. Glover, Thomas Dahlgren, Katrine Worsaae

**Affiliations:** 1 Marine Biological Section, Department of Biology, University of Copenhagen, Helsingør, Denmark; 2 Plant Functional Biology and Climate Change Cluster, Department of Environmental Science, University of Technology Sydney, Sydney, Australia; 3 Scripps Institution of Oceanography, University of California San Diego, San Diego, California, United States of America; 4 Zoology Department, The Natural History Museum, London, United Kingdom; 5 Uni Environment/Uni Research, Bergen, Norway; University of Bergen, Norway

## Abstract

Members of the conspicuous bone-eating genus, *Osedax*, are widely distributed on whale falls in the Pacific and Atlantic Oceans. These gutless annelids contain endosymbiotic heterotrophic bacteria in a branching root system embedded in the bones of vertebrates, whereas a trunk and anterior palps extend into the surrounding water. The unique life style within a bone environment is challenged by the high bacterial activity on, and within, the bone matrix possibly causing O_2_ depletion, and build-up of potentially toxic sulphide. We measured the O_2_ distribution around embedded *Osedax* and showed that the bone microenvironment is anoxic. Morphological studies showed that ventilation mechanisms in *Osedax* are restricted to the anterior palps, which are optimized for high O_2_ uptake by possessing a large surface area, large surface to volume ratio, and short diffusion distances. The blood vascular system comprises large vessels in the trunk, which facilitate an ample supply of oxygenated blood from the anterior crown to a highly vascularised root structure. Respirometry studies of *O. mucofloris* showed a high O_2_ consumption that exceeded the average O_2_ consumption of a broad line of resting annelids without endosymbionts. We regard this combination of features of the respiratory system of *O. mucofloris* as an adaptation to their unique nutrition strategy with roots embedded in anoxic bones and elevated O_2_ demand due to aerobic heterotrophic endosymbionts.

## Introduction

The unique bone-eating organism, *Osedax* (Siboglinidae, Annelida) was first described in 2004 from a whale fall located at 2891 m depth in Monterey Bay, Pacific Ocean [1]. Since its first discovery it has been found on multiple whale falls in the Pacific and Atlantic Oceans, artificially deployed cow bones [2] as well as on other vertebrate bones such as those of teleost [3]. There are five formally described species, with at least a further 12 species known from genetic evidence [4], [5]. In addition, convincing fossil traces of *Osedax* have been found in Oligocene and Pliocene mammal bones [6], [7].

**Figure 1:**
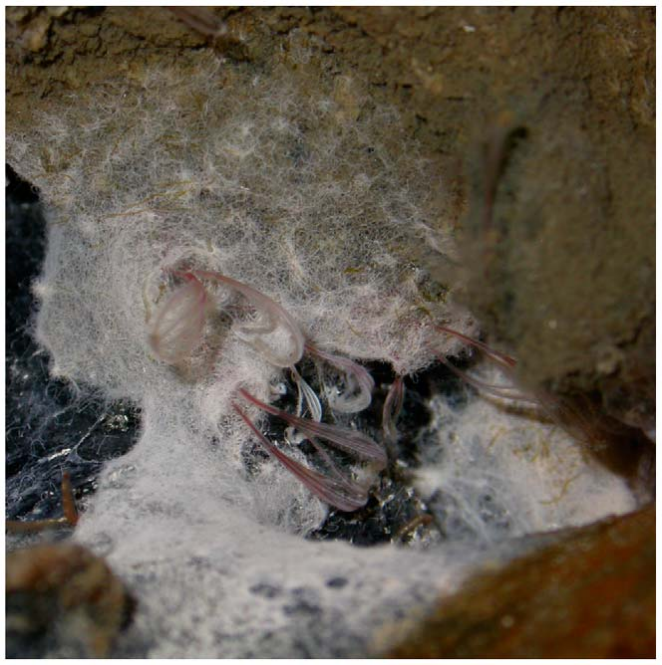
*Osedax mucofloris* penetrating a bacterial mat resembling *Beggiatoa*. *Beggiatoa* live in the restricted interface between hydrogen sulphide presence and oxygenated water [51]. *O. mucofloris* must therefore be in contact with toxic sulphide concentrations. **Photographer:** Helena Wiklund, Department of Zoology, Göteborg University, Sweden.

Evidence suggests that *Osedax* females utilize the complex organic compounds of the bone through endosymbiotic aerobic heterotrophic bacteria (Oceanospirillales) located in bacteriocytes in a root system that is embedded in the bone matrix [8], [9]. The O_2_ microenvironment around the embedded root system of *Osedax* has not been studied, yet this knowledge is crucial for understanding the function of *Osedax* in its natural habitat. The O_2_ supply within the bone matrix is presumably strongly diffusion limited, and with whale bone lipid content reaching 45% in Sei whales (closely related to Minke whales) [10] the bone interior may become O_2_ depleted due to high heterotrophic microbial activity, including sulphur-reducing bacterial processes that generate sulphide, further reducing O_2_ below the bone surface. External O_2_ supply to the roots of *Osedax* via the bone matrix is thus highly unlikely. Yet, symbiosis with detoxifying sulphide-oxidizing bacteria has so far not been proven, though *Osedax* has been observed along with sulphophilic species and mats of white *Beggiatoa*-resembling bacteria (Figure 1) or bones exhibiting ferrous sulphide precipitation [11]–[14]. The highly folded epidermis of the root structure of *Osedax* ‘green palp’ has recently been shown to lack a cuticle and possess an extensive microvillous border [15], potentially facilitating the uptake of organic substrates, but also facilitating interaction with sulphide from the root surroundings. In the methane seep-dwelling vestimentiferan *Lamellibrachia* the sediment-embedded posterior body region (also called root) is actually the main source of hydrogen sulphide uptake, and is used for maintaining the chemoautotrophic endosymbionts [16], [17]. Respiratory features in other siboglinids involve ciliary or muscular ventilation of the chitinous tube and expanded branchial structures with extensive blood vascular systems and large respiratory surface areas [16], [18]–[20]. Whereas the tube of *Osedax* is gelatinous and only covers the trunk, the vascularized and pinnulated palps of *Osedax* are believed to have a similar function as the anterior branchial plumes of Vestimentifera [21]. However, neither these structures nor the blood vascular system have been examined in detail, nor has the O_2_ consumption been measured previosly in any *Osedax* species.

*Osedax* and Vestimentifera are tubicolous species and apart from the ability to retract into their tubes when disturbed, they show very little motile activity. However, *Riftia pachyptila* has been shown to metabolize much faster than other resting annelids (e.g. double the O_2_ demand of *Arenicola marina* Lamarck, 1801) [22]. Moreover, Childress et al. [22] apparently measured the O_2_ uptake without the presence of sulphide, thereby underestimating the O_2_ consumption of chemosynthetic endosymbionts. Later, Girguis & Childress [23] showed that O_2_ uptake was reduced significantly (from 14.35±1.23 µmol O_2_ g^−1^ wet wt h^−1^ to 2.88±0.89 µmol O_2_ g^−1^ wet wt h^−1^) when the activity of endosymbionts was restricted by blocking sulphide supply. This clearly demonstrated that the metabolism of the endosymbionts of *R. pachyptila* contribute significantly to its O_2_ consumption. Experiments by Freytag et al. [17] further confirms this as an increase in O_2_ consumption of ∼42% was seen when *Lamellibrachia* cf. *luymesi* was exposed to hydrogen sulphide at the root area. The increase in O_2_ consumption is a result of the activation of the metabolism of chemoautotrophic endosymbionts. Since *Osedax* also carries aerobic endosymbionts and furthermore is hypothesised to be partly exposed to hypoxia or anoxia, knowledge of the O_2_ uptake of *Osedax* as compared to *R. pachyptila* and other annelids is relevant for enhancing our understanding of the respiratory systems and physiology of *Osedax*.

The shallow water species *Osedax mucofloris* Glover, Källström, Smith, Dahlgren, 2005, has been found at 30 m and 125 m water depth off the coast of Tjärnö, Sweden [12], [13], and at 120 m depth in Bjørnafjord, Norway [24]. Currently these are the only records of *Osedax* from the Atlantic, though other species are found in shallow waters in both the East and West Pacific [4], [25]. The 125 m deep locality near Tjärnö is characterized by stable oceanic salinity (34–35‰), temperature (5–7°C) and dissolved O_2_ concentration ranging from 4.6 to 6.3 ml O_2_ l^−1^ [13].

In the present study we investigated whether *Osedax mucofloris* is exposed to an inhospitable hypoxic or anoxic microenvironment by measuring the O_2_ distribution surrounding the embedded root system. We assessed possible morphological and/or physiological adaptations to the environmental conditions through detailed morphological studies of the respiratory surfaces and the blood vascular system as well as respiratory measurements of O_2_ consumption. The results are discussed in relation to the unique environment, endosymbionts, and embedded root structure of *Osedax* as well as compared with studies of related annelids.

## Results

### Ventilating mechanisms

#### Ciliary bands and sensory structures (anti acetylated α-tubulin staining and histology)

In the description below, we use a dorsal-ventral definition opposite to the one used by Rouse et al. [1], [21], based on a new interpretation (Rouse & Worsaae, unpublished).

**Figure 2:**
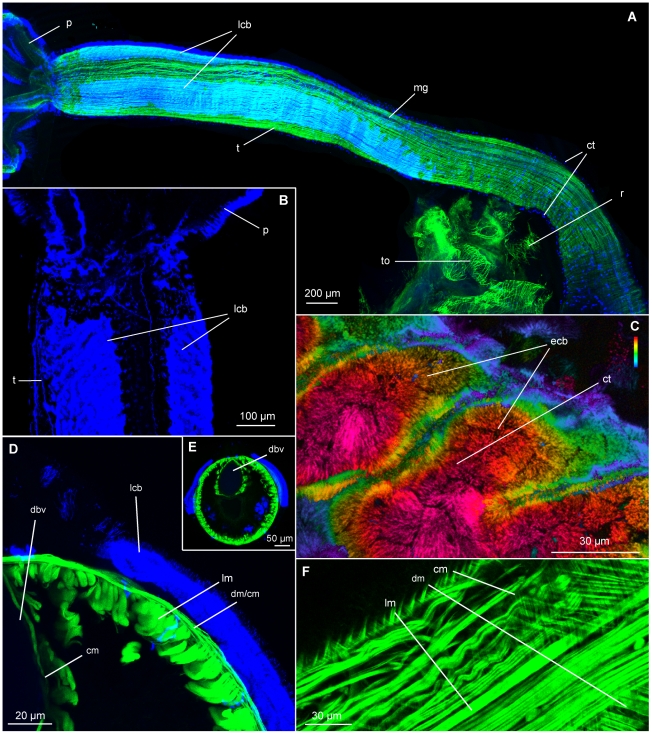
Confocal laser scanning microscopy (CLSM) of the trunk of *Osedax mucofloris* females. A: Dorso-lateral view of a complete specimen, lateral ciliary bands occupies half the length of the trunk. B: Dorsal view of the anterior part of the trunk, elliptical shaped cilia bundles are directed anteriorly from the lateral part of the trunk. C: Depth coded z-stack, the elliptical shaped cilia bundles constituting the lateral ciliary band are formed by ciliary tufts. D: Close-up of transverse section of a trunk. E: Transverse section of a trunk, note the muscularized dorsal blood vessel. F: Single z-stack image of the trunk musculature, longitudinal muscles beneath circular and diagonal muscles. Abbreviations: ciliary tufts (ct), circular muscles (cm), diagonal muscles (dm), elliptical ciliary bundles (ecb), lateral ciliary band (lcb), longitudinal muscles (lm), muscular gap (mg), palp (p), root structure (r), torn ovisac (to), trunk (t), dorsal blood vessel (dbv).

We found two previously unreported broad longitudinal ciliary bands dorso-laterally on each side of the oviduct on the anterior part of the trunk (Figure 2A, B, E). The ciliary bundles of the bands consist of multi ciliated cells, appearing pillow-like with numerous, conspicuously short cilia (<10 µm long) projecting outwards from the centre of the elliptical bundles (Figure 2C). The bundles are organized in an anterior-dorsal diagonal pattern within the longitudinal bands (Figure 2B). The ciliary bands narrow toward the posterior part of the trunk and are replaced by single tufts of cilia scattered basally across the trunk surface (Figure 2A). The short cilia appeared immotile, and with several longitudinal nerves running beneath, (Worsaae & Rouse, unpublished) their function may be sensory rather than ventilatory.

**Figure 3:**
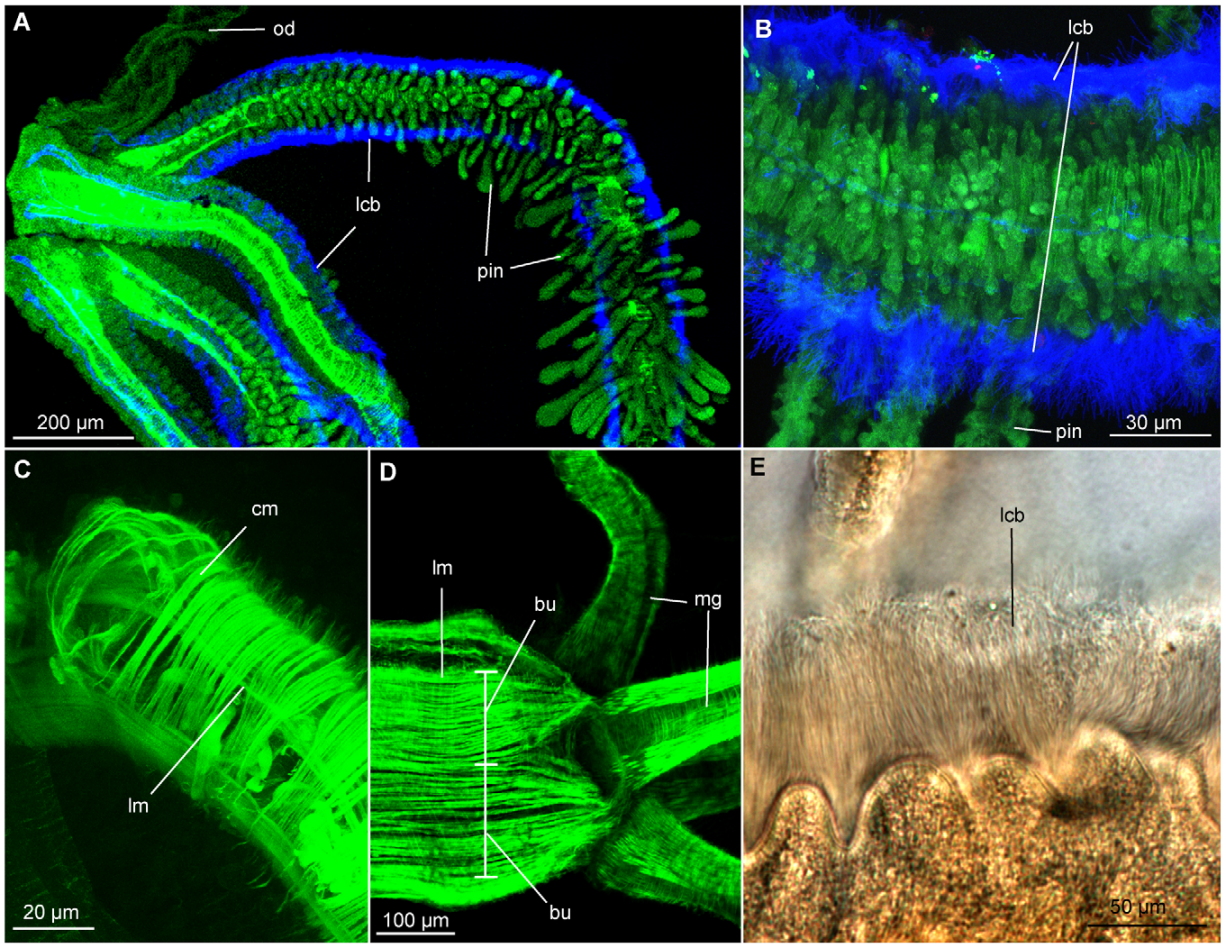
CLSM (A–D) and differential interference contrast (DIC) light micrographs (E) of the anterior palps and pinnules of *O. mucofloris* females. A: Lateral view of palps, pinnules increasing in length and development along the palp. B: Abfrontal view of the midsection of a palp, ciliary bands on each side. C: Close-up of palp musculature. D: Lateral view of the muscular palp-trunk connection. E: Close-up of the lateral ciliary band of a palp. Abbreviations: circular muscles (cm), lateral ciliary band (lcb), longitudinal muscles (lm), muscular bundles (bu), muscular gap (mg), oviduct (od), pinnules (pin).

**Figure 4:**
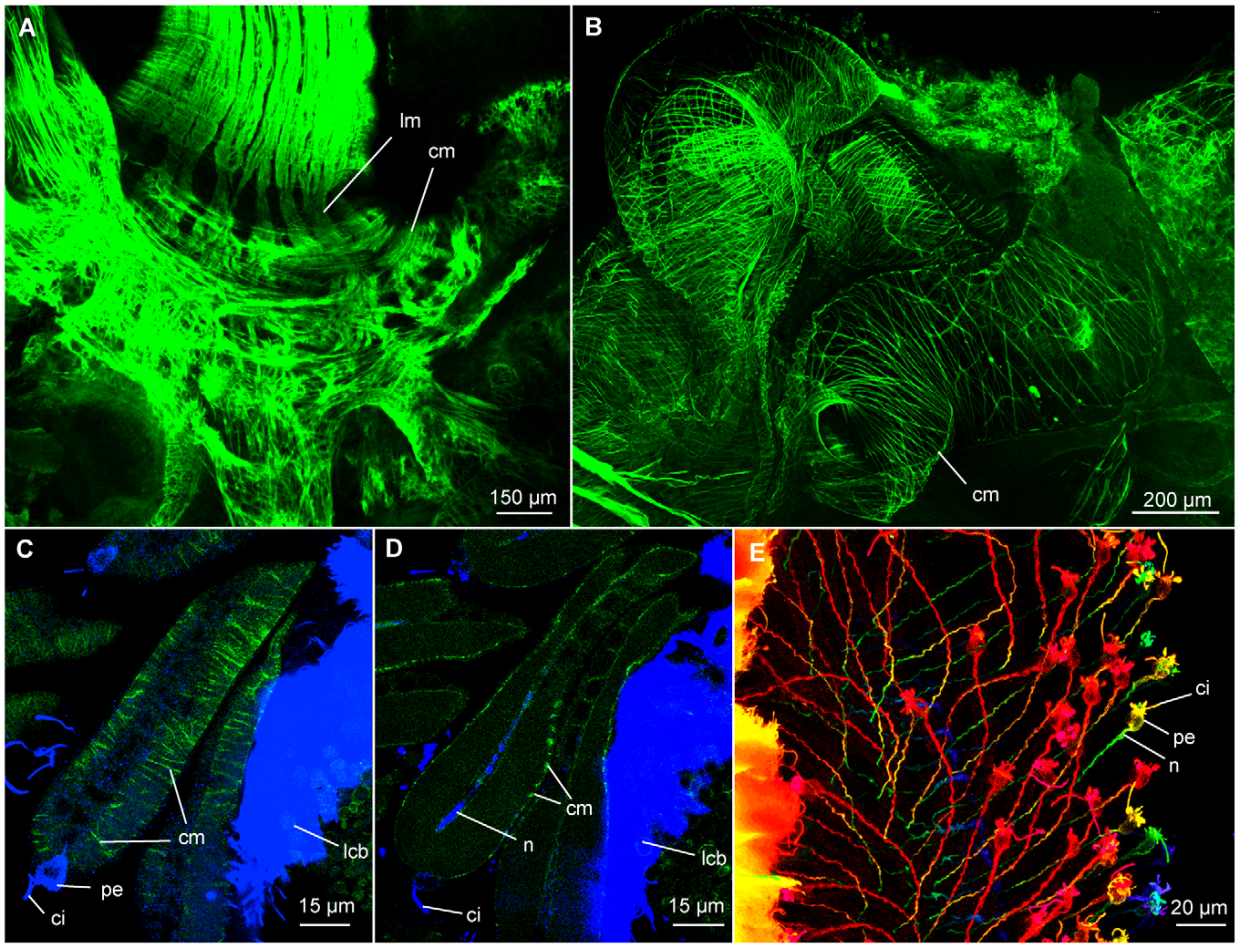
CLSM of the root system and pinnules of *Osedax mucofloris* females. A: Single z-stack image of the ‘trunk-root system’ connection, note the bundles of longitudinal muscles. B: Musculature located by the ovisac, assumed to be the posterior end of the longitudinal dorsal blood vessel. C: Single z-stack image of pinnules, circular musculature encircling the pinnular loop, note the distal perikaryon and sensory cilia. D: Single z-stack image showing a longitudinal section of the pinnule in C, note the internal nerve. E: Depth coded z-stack, pinnule nerves in *Osedax* ‘yellow-collar’. Abbreviations: cilia (ci), circular muscles (cm), lateral ciliary band (lcb), longitudinal muscles (lm), nerve (n), perikaryon (pe).

**Figure 5:**
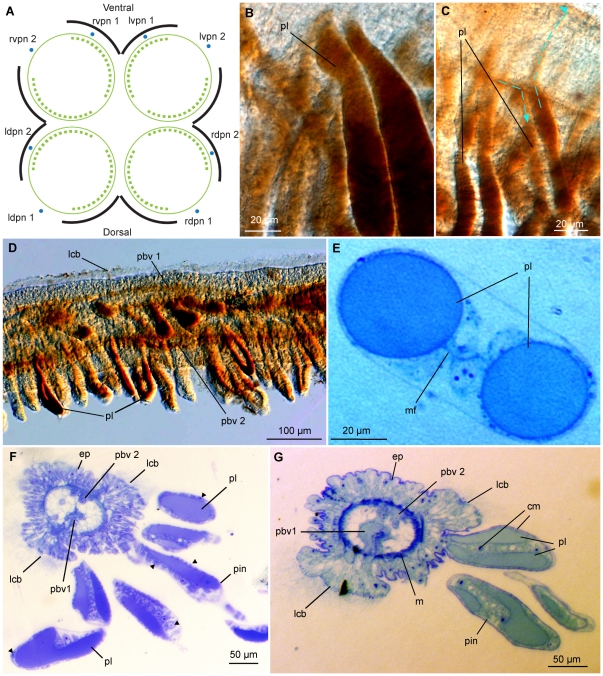
Diagram of a transverse section of the basal part of palps (A), DIC light micrographs of benzidine stained palps (B–D) and transverse 1.2 µm sections of *O. mucofloris* palps stained with toluidine blue (E–G). A: Diagram of a transverse section at the basal part of the palp region. Circular musculature (continued green lines) encircles the longitudinal musculature (green broken lines) in a cylinder formation. Two gaps separate the longitudinal muscle bands. Black lines illustrate the motile lateral ciliary bands and two main palp nerves run along each palp as shown (blue dots). B: Pinnular loop filled with blood. C: Pinnular loops, broken lines and arrows indicate the assumed direction of blood flow. D: Midsection of palp, longitudinal blood vessels and pinnular loops visible. E: Transverse section of a pinnule, the pinnular loop enclosed by a membrane fusing in the centre. F: Transverse section of the distal part of a palp, arrow tips shows circular musculature. G: transverse section of the distal part of a palp, the two longitudinal blood vessels obvious. Abbreviations: circular muscles (cm), epidermis (ep), lateral ciliary band (lcb), left dorsal palp nerve (ldpn), left ventral palp nerve (lvpn), membrane fusion (mf), musculature (m), palp blood vessel (pbv), pinnule (pin), pinnular loop (pl), right dorsal palp nerve (rdpn), right ventral palp nerve (rvpn).

Two dense ciliary bands are found on each palp along their epidermal longitudinal lobes on each lateral side, as previously reported [12] (Figures 3A, B, E, 4C, 5A, F, G). The bands consist of multi ciliated cells with 70–90 µm long cilia (Figure 3E). The ciliary bands extend from near the basal part of the palps to the distal tip, beating in metachronal waves.

Two nerves, originating at the anterior part of the brain, innervate each palp. One nerve runs abfrontally, between the two lateral ciliary bands and the other nerve runs laterally underneath one of the ciliary bands (Figure 5A). Further details on the female *Osedax* nervous system will be described elsewhere (Worsaae & Rouse, unpublished).

Pinnules (up to 100 µm wide) project perpendicularly from the frontal palp surface between the lateral ciliary bands, with a density of approximately eight pinnules across the palp per 50 µm palp length (Figures 3A, 5F, G). At the proximal end of the palps, pinnules are less developed than at the distal end, seemingly growing in length (up to 170 µm) synchronously with the growth of the palp (Figure 3A). A sensory cell extends through the centre of the pinnule with a distal perikaryon and a few external, presumably sensory, cilia (Figure 4C, D). Its axon seems to connect with one of the two major longitudinal palp nerves, possibly the one running more laterally beneath the ciliary band. This is supported by similar findings in *Osedax* “yellow-collar" (Figure 4E).

#### Musculature (F-actin, phalloidin staining)

The longitudinal muscles run along the entire length of the trunk (Figure 2A), originating posteriorly at the trunk basis, and inserting anteriorly at the base of the four palps (Figure 3D). In one individual, it was possible to detect clustering of longitudinal muscles into 14–16 bundles of >20 muscle strands in the posterior part of the trunk, anterior to the root structure (Figure 4A). Along, and around the entire trunk, the muscles are distributed in a dense cylindrical formation with an average of six strands per 50 µm (Figures 2D, E, 3D). The musculature is slightly separated internally to the oviduct (possibly by the nerve cords), as well as randomly along the trunk, creating minor gaps most likely for mucus gland exits or nerves (Figures 2A, 3D). Anteriorly, a gap in the musculature is found ventrally at the position of the brain, as well as at each of the four insertion points of the palps. At each of these four points, the longitudinal musculature divides into two bundles (of each 20–30 strands), encircling the insertion point of the palp (Figure 3D). The longitudinal palp muscles originate at the base and run along the entire length of the palps to their tips, separated by a smaller frontal and a larger abfrontal gap (Figures 3C, D, 5A). No longitudinal musculature was detected in the pinnules.

Around the ovisac and anterior root structure the longitudinal musculature divides, and together with the circular muscles, creates a mesh-like structure (Figure 4A). The musculature extends posteriorly along the roots in a cylindrical formation, supporting the tissue penetrating the bone.

Thin circular muscles are found peripheral to the longitudinal muscles along the entire trunk, with 24 strands per 50 µm (Figure 2D). The circular muscles are most dominant in the posterior part of the trunk and continue into the root structure (Figure 4A). Notably, much thicker diagonal muscle strands were found beneath the circular musculature, but peripheral to the longitudinal muscles (Figure 2F). Attached at the mid-dorsal line, the strands run diagonally around the trunk in an anterior direction and attach at the mid-ventral line. The diagonal muscles are distributed along the entire length of the trunk, lying further apart (35–70 µm) in the posterior end than along the rest of the trunk (5–10 µm).

The circular musculature of the palps encircles the longitudinal musculature as a cylinder with ∼18 muscle strands per 50 µm and is evenly distributed along the entire palp (Figure 3C). Fine circular muscles enclose each vessel of the pinnular loop (Figure 4C, D); they were likewise visible in the semi-thin sections on the outside of the pinnular loop and along the midline of each pinnule (Figure 5F, arrow tips), with a spacing corresponding to those shown with phalloidin staining (Figure 4C, D).

### Blood vascular system

**Figure 6:**
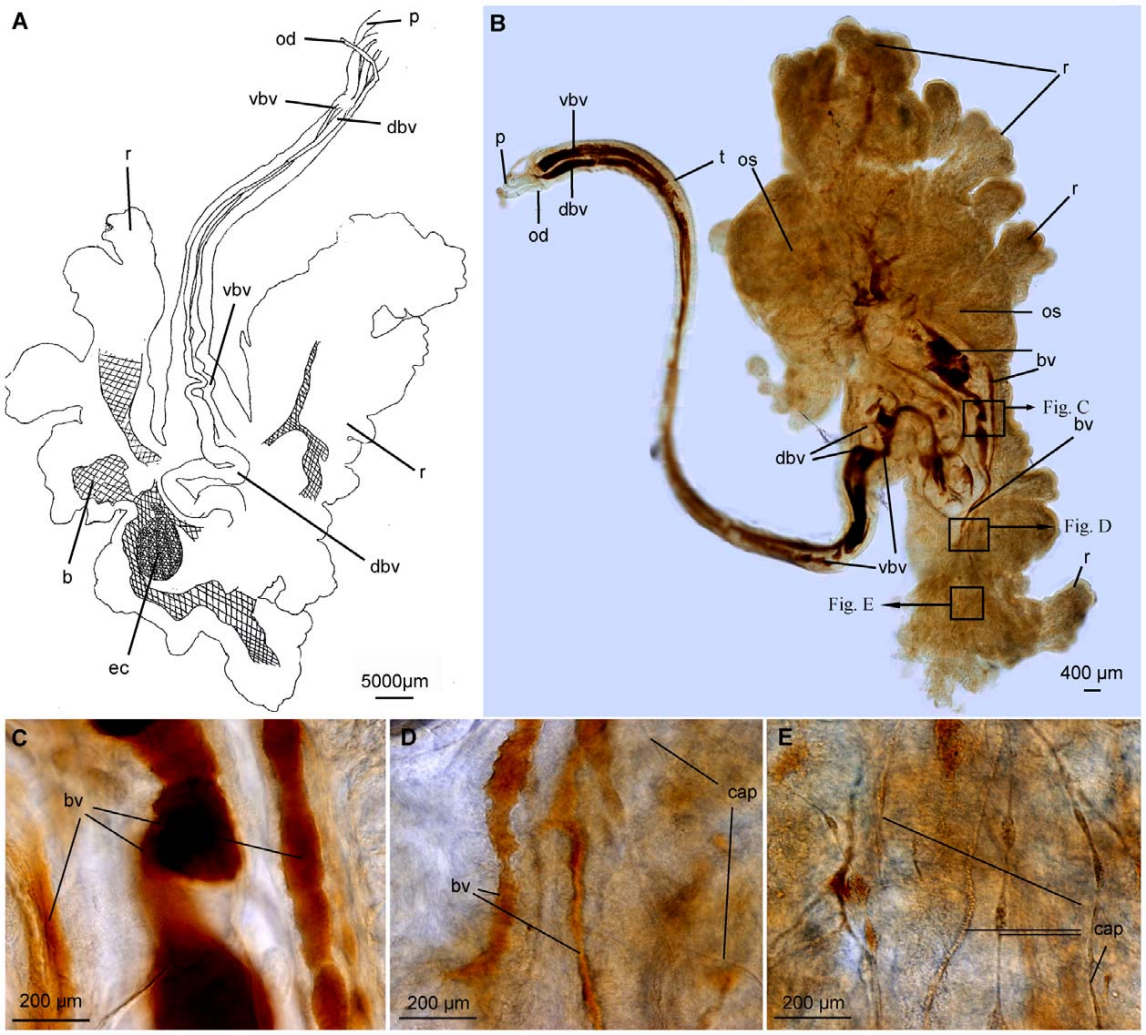
DIC light micrographs of benzidine stained *Osedax mucofloris*. A: Sketch of the path of the longitudinal trunk vessels into the root structure, drawn from the light microscope with a camera lucida of a benzidine stained *O. mucofloris* female. Trunk twisted in midsection. B: DIC light micrograph of a benzidine stained *O. mucofloris* female. Lateral view, trunk twisted in midsection. Ventral and dorsal blood vessels continues, folded, into the anterior part of the ovisac/root system. C: close up of blood vessels near ovisac. D: close up of blood vessels supplying more distally placed capillaries. E: Capillaries supplying tissue and endosymbionts. Abbreviations: blood traces (b), blood vessel (bv), capillaries (cap), dorsal blood vessel (dbv), egg cluster (ec), oviduct (od), ovisac (os), palp (p), root structure (r), trunk (t), ventral blood vessel (vbv).

The trunk of *Osedax mucofloris* encloses two major longitudinal blood vessels (Figures 2E, 6A, B). The dorsal vessel is highly muscularized with circular musculature throughout its length (Figure 2D, E). No lateral connecting vessels between the major longitudinal vessels or epidermal capillaries were found in the trunk. From the trunk, the two blood vessels continue posteriorly into the root structure and increase in diameter (Figure 6A, B). When studied under the light microscope, the muscularized dorsal vessel is visible as a defined tube along the trunk, and continues into the anterior root structure, curling up in the centre anterior to the ovisac. The curling configuration is most likely caused by contraction of the basal part of the trunk. The exact further path of the vessel was difficult to determine. The Confocal Laser Scanning Microscope (CLSM) studies also showed the continuation of the dorsal blood vessel around the ovisac, revealing cylindrical musculature at the ovisac, corresponding in diameter to the musculature of the dorsal blood vessel of the trunk (Figure 4B).

The ventral blood vessel also continues into the root structure, but as a narrower and less defined vessel, the path of which was even more difficult to determine, than that of the dorsal vessel. Blood vessels of different sizes, as well as multiple obvious capillaries within the root tissue were observed (Figure 6B–E). Larger vessels extend out from the area of the ovisac and divide into thinner vessels (diameter: 7–30 µm). Capillaries were detected in the periphery of the root tissue (Figure 6E), with distances between the detected capillaries ranging from 65–220 µm.

**Figure 7:**
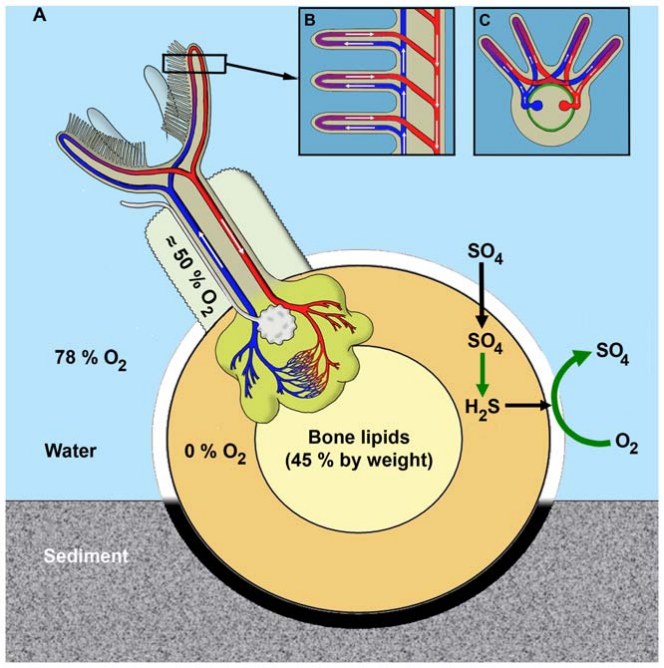
Schematic illustration of O_2_ distribution in the internal and external environments of *Osedax mucofloris*. A: *Osedax mucofloris* extend its palps and pinnules, with large respiratory surfaces, into the overlying O_2_-rich water in order to uptake O_2_. O_2_ is then distributed to the buried root system through the extensive blood vascular system also supplying the heterotrophic endosymbionts. The O_2_ distribution to the root system is crucial as local uptake is not possible in the anoxic bone environment. The anoxic environment is partly produced by intense bacterial processes (green arrows) utilizing O_2_ at the bone surface. Hydrogen sulphide is produced by anoxic bacterial processes within the bone matrix during decomposition of organic content using sulphate. B: Schematic illustration of assumed blood flow in palp and pinnules, longitudinal section. Blue vessels carrying venous blood through afferent vessels, red vessels carrying arterial blood through efferent vessels. C: Schematic illustration of assumed blood flow in palp and pinnules, transverse section. Likewise blue vessels carries venous blood through afferent vessels, red vessels carries arterial blood through efferent vessels. Note that the palp blood vessels are created by an invagination of the basement membrane. Green indicates musculature.

Anteriorly, each palp encloses a pair of blood vessels created by invaginations of the inner lamina of the basement membrane of the epidermis (Figures 5F, G, 7C). Imaging of live specimens confirmed the presence of two blood vessels, running along the entire length of the palps.

The pinnules are largely filled by a blood cavity lined with a membrane, which fuses in between the two blood cavities along most of the pinnule length, thereby creating the pinnular loop (Figures 5B–G, 7C). The palps of the histological sections were ∼300 µm in diameter at the base and the palp epidermis was ∼50–75 µm thick. The palp diameter and the thickness of the epidermis were both found to decrease towards the distal end of the palp.

Pinnules vary in diameter along their length and along the palp, with a median diameter of ∼40×100 µm for the pinnules and ∼20–40 µm for each blood vessel. The diffusion distance across the pinnule epithelium was measured on the semi-thin sections to be 1–2 µm, and for the epidermis of the distal part of the palps, the diffusion distance was measured to be ∼30 µm.

### Oxygen consumption

When corrected for background respiration, the measured O_2_ consumption (MO_2_) of *O. mucofloris* ranged almost across a factor of ten from 220±93 µg O_2_ g^−1^ h^−1^ to 2053±1950 µg O_2_ g^−1^ h^−1^ (Table 1), depending on the approach used for measurement. MO_2_ measured on *O. mucofloris* inhabiting sectioned bone pieces (B1–B3) vs. MO_2_ measured *O. mucofloris* inhabiting bones in cuvettes (C1, C2), resulted in two distinctly different ranges of MO_2_. The MO_2_ of C1 and C2 was 220±93 µg O_2_ g^−1^ h^−1^ and 238±29 µg O_2_ g^−1^ h^−1^, respectively, while the MO_2_ of B1–B3 range from 976±201 µg O_2_ g^−1^ h^−1^ to 2053±1950 µg O_2_ g^−1^ h^−1^. Methods and ranges are commented further in the discussion.

**Table 1:** Weight specific O_2_ consumption (MO_2_) of *Osedax mucofloris*.

	Numbers of individuals	Fixed weight (g)	MO_2_ (µg O_2_ g^−1^ h^−1^)	SD^a^	N^b^
B1	4	0.081	976	201	3
B2	4	0.044	1292	650	6
B3	1	0.017	2053	1950	3
C1	2	0.054	220	93	5
C2	3	0.11	238	29	7

MO_2_ was determined in sea water at 100% atmospheric saturation and carried out on *O. mucofloris* inhabiting sectioned cow bone (B1–B3) and cow bone in cuvettes (C1–C2).

a Standard deviation.

b numbers of measuring sequences on which MO_2_ is based.

### Oxygen levels around *Osedax*

**Figure 8:**
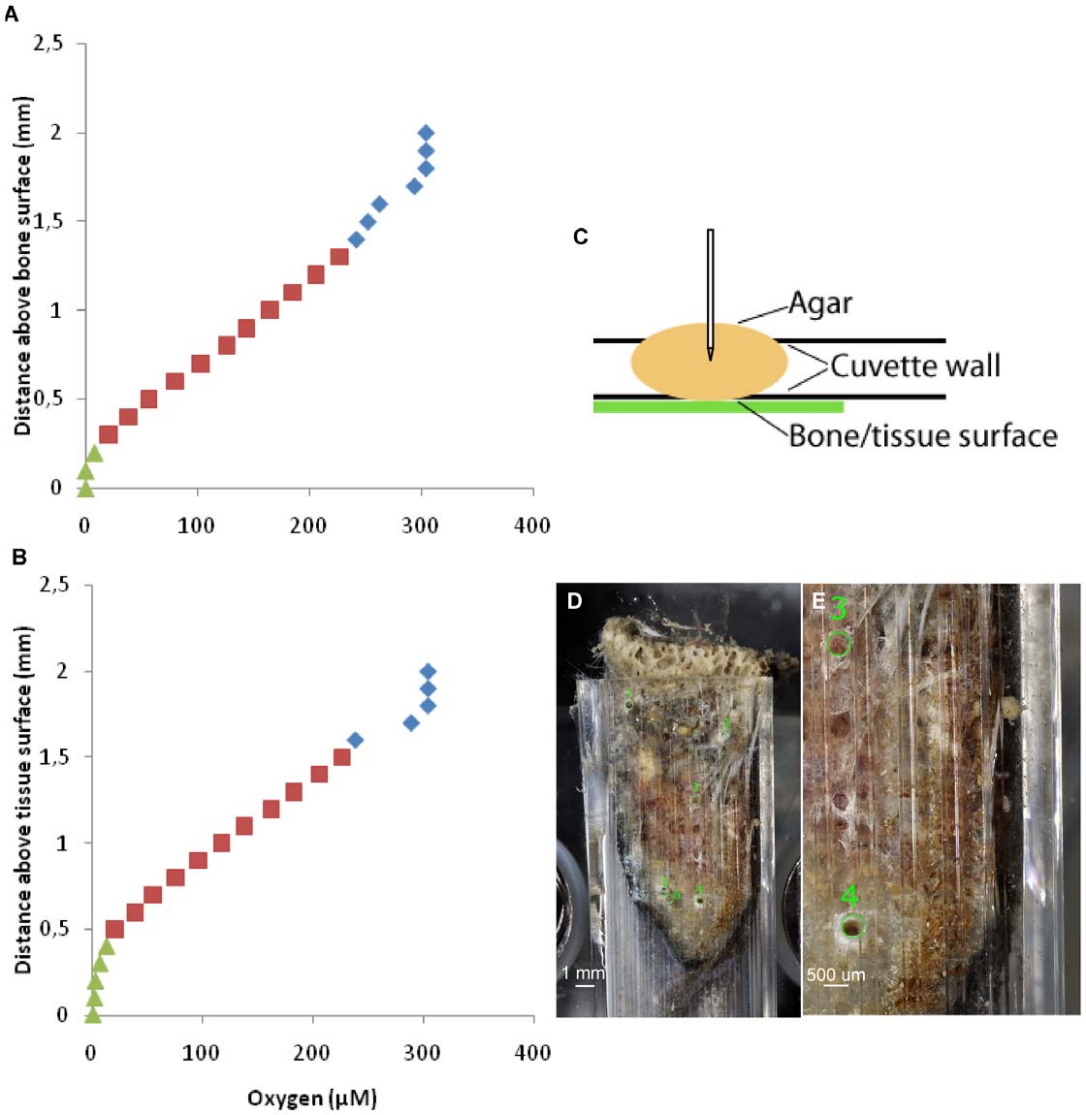
Experimental setups of micro sensor measurements and O_2_ profiles towards bone and tissue surfaces. A: Depth profile of O_2_ towards bone surface, blue: agar, red: cuvette wall, green: bone surface. B: Depth profil of O_2_ towards tissue surface, blue: agar, red: cuvette wall, green: tissue surface. C: Schematic drawing of the micro sensor measuring path through the cuvette wall. D: Placement of measuring site on WB1, note the blackened areas indicating presence of ferrous sulphide. E: Close-up of measuring sites on WB1.

Micro sensor measurements of O_2_ concentrations in proximity to the bone interface were conducted, through agar-filled holes, on *Osedax*-colonized bone fragments in cuvettes. The obtained profiles of O_2_ concentration from the aerated seawater, and inwards showed steep O_2_ gradients towards both the bone and tissue surface (Figure 8A, B). Anoxic conditions or very low O_2_ levels were found at the bone surface, within the bone, and in proximity to the embedded root tissue of *O. mucofloris* (Table 2). The average O_2_ flux at the bone and tissue interface was 0.028±0.0024 nmol O_2_ cm^−2^ s^−1^ (n = 5) and 0.029±0.0040 nmol O_2_ cm^−2^ s^−1^ (n = 3), respectively.

**Table 2:** O_2_ concentration measured by micro sensors at the bone or tissue surface of *Osedax mucofloris* inhabiting whale bone in two cuvettes, WB1 and WB2.

Bone surface	O_2_ concentration (µmol l^−1^)	
	Site 1	0.00
	Site 2	0.00
WB 1	Site 4	0.00
	Site 5	0.00
	Site 6	0.00
	Site 1	0.00
WB 2	Site 2	0.00
	Site 5	3.44
Tissue surface		
WB 1	Site 3	0.00
WB 2	Site 3	0.00
	Site 4	0.67

Micro sensor measurements of O_2_ distribution in one mucus tube showed a ∼50% decrease in the O_2_ concentration in the centre of the mucus tube wall as compared to outside the tube (Table 3). Direct measurements within the tube were not possible due to disturbance by the worm. Measurements of the O_2_ microenvironment surrounding the palps showed a strong decrease in O_2_ concentrations, when a palp approached the microelectrode measuring tip. At the base and middle of the palp, the O_2_ levels were almost zero showing the O_2_ uptake to be high in these areas (Table 3). At the distal end of the palp the O_2_ concentration was only reduced to approximately 50% atmospheric saturation, possibly due to decaying palp tips.

**Table 3:** O_2_ concentration measured by micro sensors in a mucus tube wall and at the epidermis of palps of *Osedax mucofloris*.

Mucus tube wall		Palp	
Site	O_2_ concentration (µmol l^−1^)	Site	O_2_ concentration (µmol l^−1^)
Free water	297.86	Base I	0.00
Surface	221.94	Base II	0.57
Breached tube	122.65	Middle	5.73
Centre	170.54	Tip	148.99

## Discussion

### Ventilation and branchial structures

The presence of highly vasculated palps and pinnules, the former densely ciliated showed that the anterior crown is the main site for O_2_ uptake (Figure 7A–C). Uptake of O_2_ over the trunk surface is possible, but a thicker epidermis, short and seemingly immotile ciliary bands and no obvious respiratory structures suggest that the trunk is a minor site of O_2_ uptake. Oxygen does, to some extent, diffuse from the surrounding water into the mucus tube, thereby supplying the dwarf males.

The well-developed longitudinal musculature of the trunk serves to retract the trunk and palps into the tube and bone, presumably as protection from predators. As no regular retraction patterns of trunk musculature were detected, retraction into the tube is not considered a significant mode of ventilation for required O_2_. Furthermore, the tube only surrounds the trunk, tightly fitting to the base of the trunk and the surface of the bone, preventing any water exchange to the ovisac and roots from the bone surface. The longitudinal musculature is moreover able to stretch the extensive branchial structures (palps) into the aerated water in order to increase the O_2_ uptake.

The circular trunk musculature in *O. mucofloris* is weakly developed, as found in several other annelids [26], [27]. The diagonal musculature revealed in the present study, may compensate for the weak circular musculature by having a similar supportive function. Diagonal musculature has most likely been mistaken for circular musculature in several annelids [26], [27], including previous studies of *Osedax* [15], [21].

Our study demonstrates the respiratory challenges faced by *Osedax*, as no oxygen was measured in the bone matrix as well as in the immediate vicinity of root structures and ovisac. Furthermore, we found no ventilating structures such as ciliary bands on the root epidermis, nor dense capillaries beneath the epidermis. Therefore, the epidermis of the embedded root structure is unlikely to facilitate ventilation or local O_2_ supply to the *Osedax* root system. The O_2_ necessary to maintain the metabolism and heterotrophic endosymbionts of the roots may instead primarily be transported via the blood vascular system from uptake at the aerated palps (Figure 7A). However, the microvillar root epidermis in *Osedax* does constitute a large surface area to volume ratio, which besides uptake of organic compounds may also facilitate uptake of e.g., hydrogen sulphide in the bone matrix. This is the case for the ‘root’ surface of the vestimentiferan *Lamellibrachia*, where hydrogen sulphide is absorbed and then transported via the circulatory system to the chemoautotrophic endosymbionts in the trophosome [16], [17], [28].

Previous studies of other close relatives of *Osedax* such as Vestimentifera and Sabellidae have shown anterior branchial structures to be of vital importance when tube-bound tissue is not ventilated [29]–[31]. Likewise, the anterior branchial structures of *O. mucofloris* seem morphologically adapted to facilitate efficient O_2_ uptake form the surrounding water. As with the branchial plume of Vestimentifera [31], [32], the anterior palps and pinnules of *O. mucofloris* have a large surface area to volume ratio and short diffusion distances. Furthermore, O_2_ uptake is optimized by the two ventilating ciliary bands on each palp, a feature also seen in other Siboglinidae and in Sabellida in general [20], [31], [33].

A rough estimate was made of the surface area of the anterior crown, calculated from palp length given by Glover et al. [12], and the dimensions of palps and pinnules of *O. mucofloris* found in the present study. This results in a weight specific branchial surface area (SBSA) of ∼22 cm^2^ g^−1^ fixed mass, which is similar to the SBSA of *Riftia pachyptila* and higher than the SBSA of fish, crabs and other annelids [31]. This is especially obvious when compared to the SBSA of e.g., *Arenicola marina* (4.00 cm^2^ g^−1^) [34], although this species can also take up O_2_ across its general epidermis.

The estimated diffusion distance in *O. mucofloris* (pinnule epidermis 1–2 µm, palp epidermis ∼30 µm) is furthermore comparable to what has been found for *R. pachyptila* (pinnules ∼2.00 µm; branchial filaments ∼25 µm) and *Ridgeia piscesae* (pinnules ∼1.00 µm; branchial filaments ∼17 µm) [31], [32].

Interestingly, the present study showed that each pinnule is equipped with a distal sensory cell and external sensory cilia. These structures may sense disturbance in the water to avoid predators, as *Osedax* need not sense food items or reproductive indicators in the water [1], [8], [35]. Additionally, sensing of currents may also be beneficial in order to orientate the palps e.g., for optimal uptake of dissolved O_2_. *Riftia pachyptila* does not have sensory structures on the pinnules, but does have long, separate sensory filaments that lack ciliary bands and pinnules. These structures, with unknown function, are placed between pinnulated filaments [33].

### Blood vascular system

The quantity and complexity of capillaries in the root structure of *O. mucofloris* reflects the high O_2_ demand of *Osedax*, presumably for the metabolism of its heterotrophic endosymbionts and production and development of eggs. Similar capillaries are visible on the exposed ovisac of *O. frankpressi* (figure 2F in [1]) and *O. roseus* (figure 4E in [21]), while the present study shows the presence of capillaries supplying the more distally placed root tissue and bacteriocytes. However, the extent of capillaries does not match the extensive capillary network of the trophosome in other Siboglinidae [20], [33]. This is in accordance with the original description [1] mentioning the lack of a discrete trophosome, the *Osedax* trophosome instead being diffuse and beneath the epidermis of the embedded tissue.

The present study supports the basic histological findings with regard to the circulatory system of the palps in *O. roseus* [21]. The increased level of detail in the present study suggests similarities with some previous siboglinid studies [31], [32], [36]. The pinnular loop is most likely lined by an extension of the outer lamella of the basement membrane, which is in accordance with the findings of Nørrevang [36]. No indication of a capillary plexus between the two elements of the loop was observed, as otherwise seen in *Riftia pachyptila* and *Ridgeia piscesae* [31]–[33]. In accordance with Nørrevang's [36] observations of blood flow in *Siboglinum*, the blood of *Osedax* flows through an afferent vessel into the palp, through the pinnular loops and back through an efferent palp vessel (Figures 7B–C). This circulatory system carries oxygenated blood to the trunk and root system. In the present study of *O. mucofloris* and previous studies of *Osedax* [1], [21] the main trunk blood vessel has been shown to be muscularized, and is now regarded as dorsal (Rouse & Worsaae, unpublished). This is in accordance with other annelids [37] and siboglinids [20], [33], where the muscularized dorsal vessel, possibly assisted by a heart, create blood flow in an anterior direction in the dorsal trunk vessel and posterior in the ventral trunk vessel. Rouse et al. [1] reported an anterior lying dorsal heart in the original description of the genus, not noted in any descriptions since and would now appear to be an error (Rouse, pers. obs.).

The main blood flow within the palp vessels may be generated by the circular body wall musculature of the palp. However, the thin circular musculature of the pinnules surrounding each branch of the looped blood vessel (Figure 4C, D) most likely assists local blood flow, as suggested for the tentacular vessels of *Riftia pachyptila* [22]. Musculature in anterior appendages has been found in several Vestimentifera, in the form of sphincter muscles located in the branchial lamella and filament vessels [33].

As with the trunk of *O. mucofloris*, the anterior vestimentum of Vestimentifera lacks branching vessels between the ventral and the muscularized dorsal vessel [33]. The resemblance between the blood vascular systems adds new evidence to the hypothesis that the trunk of *Osedax* and the vestimentum of Vestimentifera are homologous regions [21].

### Oxygen consumption

**Figure 9:**
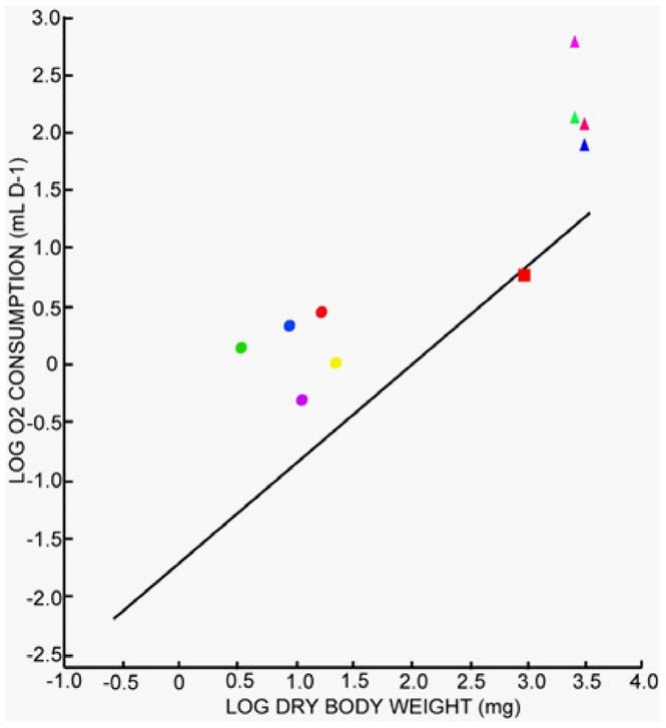
O_2_ consumption of *O. mucofloris* females set in relation to O_2_ consumption of resting annelids as well as recent data of O_2_ consumption of *R. pachyptila*. Graph modified from Cammen [18], the regression line (log R = −1.682+0.850 _*_ log W) calculated from measurements of resting nonventilating annelids only. **Dots:** B1 (red), B2 (blue), B3 (green), C1 (purple), C2 (yellow). **Triangles:** Previous measured O_2_ consumption of *R. pachyptila*. No sulphide present in water when measuring: Red, blue [22] and green [23]; sulphide present in water during measurement: Purple [23]. **Red Square:** O_2_ consumption of resting *Arenicola marina* [52].

The present study shows that *Osedax mucofloris* has a higher weight specific O_2_ consumption (MO_2_) than other resting annelids (Figure 9) [18]. This may reflect an elevated demand of the embedded tissue due to presence of heterotrophic aerobic endosymbionts, which have a higher metabolism than regular tissue. The measured MO_2_ actually corresponds to that found for *Riftia pachyptila* [22], [23] (Figure 9), possessing a vast amount of chemoautotrophic bacteria in their trophosome. Furthermore, a high MO_2_ may also reflect oxidative sulphide detoxification. The high MO_2_ corresponds well with the large SBSA and elaborate branchial structures, which are both usually associated with animals exhibiting a high O_2_ demand. This correlation is also found in *Arenicola marina* with smaller SBSA and lower MO_2_ (red square, Figure 9).

Our data show that *O. mucofloris* inhabiting bone in cuvettes (purple and yellow dots, Figure 9) have a lower MO_2_ than *O. mucofloris* inhabiting sectioned cow bone (green, blue, red dots, Figure 9). Along with high standard deviations, this highlights the difficulties of measuring O_2_ consumption on these embedded worms. The variations are most likely caused by the difference in blind respiration measurements and the large biological activity present on decaying bone, which was difficult to quantify. Future studies should thus be carried out to more precisely determine the MO_2_ of *O. mucofloris* and the contribution of other O_2_-consuming surfaces.

### *Osedax* adaptations to the bone environment

The bone matrix exhibited strong O_2_ depletion with anoxic or low O_2_ levels at its surface and with areal O_2_ consumption rates comparable to *in situ* diffusive O_2_ uptakes measured in sediments proximate to whale falls [38]. The respiratory and circulatory system of *Osedax* is well suited to these environmental challenges. The elaborate branchial structures of the palp and pinnules of *Osedax mucofloris* facilitate a high O_2_ uptake from the surrounding seawater that, in combination with the blood vascular system, apparently enables sufficient oxygenation of the embedded tissue and endosymbionts. A similar, well-developed respiratory system is also found in other members of Siboglinidae (for review see [39]), also exhibiting high surface area to volume ratio in the branchial structures in particular. Based on these morphological and functional similarities among several siboglinids it seems possible that a well-developed anterior branchial structure may be a plesiomorphic condition among siboglinids and possibly a prerequisite for housing aerobic symbionts while dwelling in anoxic habitats. This respiratory system may therefore very well be a preadaptation present in the common ancestors of *Osedax* and other siboglinids, responsible for the successful colonization and evolution of *Osedax* in the unique bone environment.

An extended root surface (and high area to volume ratio) is likewise found in both *Lamellibrachia* (e.g., [16]) and *Osedax* (yet branched). However, the main function has so far been interpreted as very different. Whereas the *Osedax* root surface is suggested to mainly facilitate uptake of organic compounds (with less focus on the possible congruent uptake of sulphide) [9], the root of *Lamellibrachia* is found to be the main respiratory surface of hydrogen sulphide necessary for the chemosynthesis of the symbionts [16], [17], [28].

The measured O_2_ depleted bone environment as well as ferrous sulphide precipitations (Figure 8E) supports the suggested exposure of *Osedax* to sulphide. Furthermore the large root surface in the related *Lamellibrachia* is highly efficient in hydrogen sulphide uptake [17]. Since the main trophic source of *Osedax* compared to other siboglinids (including *Lamellibrachia*) seems to be heterotrophic bacteria rather than sulphide-detoxifying chemoautotrophic bacteria [8], *Osedax* may instead possess physiological adaptations to detoxify sulphide.

Though beyond the scope of the present paper, microanalytical approaches such as microsensors [40], [41] and functional imaging techniques [42] could yield a more complete mapping of the chemical microenvironment of *Osedax*, including the exact levels of sulphide exposure and spatio-temporal dynamics of O_2_ in the root system. It would also be interesting to know whether *Osedax* have sulphide-binding properties of their haemoglobin as found in Vestimentifera [43], [44], as they might use them to transport toxic sulphides from the area surrounding the root structure to e.g., the palps, somehow releasing the toxic compounds to oxygenated seawater or detoxifying them.

## Materials and Methods

### Sampling and aquarium setup

**Figure 10:**
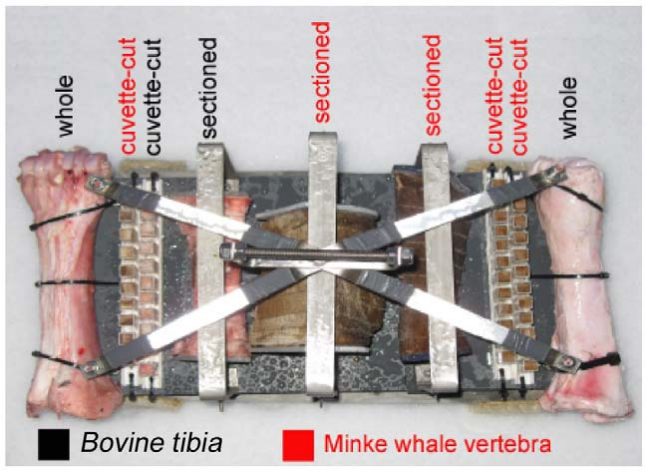
Overview of bone types in experimental sampling device for recruitment of *Osedax*. The experimental sampling devices were placed at 125 m depth off the coast of Tjärnö, Sweden (58°52.976N; 11°05.715E) in close vicinity to a minke whale carcass sunk in October 2003 [13].

In January 2009, 3 replicate experimental sampling devices, using cow and whale bone, for recruitment of female *O. mucofloris* (Figure 10) were placed at 125 m depth off the coast of Tjärnö, Sweden (58°52.976N; 11°05.715E) in close vicinity to a minke whale carcass sunk in October 2003 [13]. One device for morphological and reproductive studies was successfully retrieved in May 2009. A second device was retrieved in November 2009 for *in vivo* studies (respirometry and microsensor analysis) and additional morphological studies. Additionally, a piece of cetacean bone deposited at 123 m in the same area in May 2008 was retrieved in January 2009 and used for preliminary investigations and for designing experimental setups. In January 2009, the bottom water had a salinity of 34.6‰, a temperature of 8.4°C, and an O_2_ content of 78.7% atmospheric saturation.

Placement and retrieval were carried out with a remotely operated vehicle (ROV, Sperre Subfighter, Norway) operated from the R/V *Lophelia* (Sven Lovén Centre for Marine Science, University of Gothenburg, Sweden). Upon retrieval, the sampling device was placed in a cooling box with ambient aerated water and transported to the Marine Biological Section, University of Copenhagen within 8 hours. In the laboratory bones were kept in aerated seawater under *in situ* conditions, in aquariums (Vol 60 litre) with aeration, protein skimmers and a recirculating pumping system. The *Osedax* “yellow-collar" shown as comparison in Figure 4 was sampled in November 2009 in Monterey Bay, California at 385 m from a Grey whale skeleton and stained in accordance to the protocol described below. All necessary permits were obtained for the described whale fall experiment; received from Karin Pettersson at the County Administrative Board Västra Götalands Län, Oct. 2003. The field studies did not involve endangered or protected species.

### Fixation

Specimens were carefully dissected from the bone and fixed for immunohistochemistry, benzidine staining and histology. Prior to dissection, *Osedax mucofloris* were anesthetized for 5–10 minutes in a 1∶1 solution of seawater and MgCl_2_ (isotonic to seawater). Anaesthetized animals were gently dissected with scalpels and fixed at 4°C over night in 4% paraformaldehyde in 0.15 M phosphate-buffered saline (PBS) with 5% sucrose, pH 7.4. Subsequently, the animals were rinsed 4–6 times for 30 min in PBS with 5% sucrose and were then stored at 4°C in PBS with 0.05% sodium azide (NaN_3_).

### Immunohistochemistry

Four specimens were stained following the protocol of Worsaae & Rouse [45]. First staining included the primary antibodies monoclonal mouse anti-acetylated α-tubulin (Sigma T6793, 1∶200) & polyclonal rabbit anti-serotonin (Sigma: S5545; 1∶100/1∶400) or monoclonal mouse anti-acetylated α-tubulin & Anti-FMRFamide (ImmunoStar: 20091, 1∶100). This was complimented by secondary antibodies; anti-mouse CY5 (Jackson ImmunoResearch: 115-175-062, 1∶400) and anti-rabbit TRITC (Sigma T5268, 1∶200/1∶400). Hereafter specimens were incubated for 60 min in phalloidin conjugated with FITC or Alexa Flour 488 (Sigma F5282 or Invitrogen A12379, 0.17 or 0.33 µmol l^−1^ phalloidin in PBS). Specimens were mounted in 100% Vectashield® containing DAPI (Vector Laboratories inc., California, USA) and stored at −18°C. The specificity of primary antibody binding versus e.g., autoflourescence was tested by omitting one of the primary antibodies, but otherwise treating specimens as described.

Specimens were studied using a Leica TCS SP5 confocal laser scanning microscope (CLSM) (University of Copenhagen, Faculty of Health Science, courtesy of M. Givskov and T. Bjarnsholt). Leica LASAF computer software or Imaris® x64 6.0.0 (Bitplane AG, Zurich, Switzerland) was used to produce projections of z-stacks of CLSM images, while further analyses of z-stack series were performed with Imaris® x64 6.0.0. Computed 2D images of muscles, nerve and cilia with relation to respiration and palp morphology were further optimized with Adobe Photoshop CS3 and Adobe Illustrator (Adobe System Incorporated) for presentation.

### Histology

One specimen was embedded in epon and used for histological analysis. Semi-thick 1.2 µm sections were cut on a microtome (EM UC6, Leica, Wetzlar Germany) with a diamond knife (Diatome; Biel, Switzerland). A small amount of Pattex contact adhesive (Pattex Compact; Henkel KGaA, Düsseldorf, Germany) was diluted with a few drops of xylene in an Eppendorf tube and applied to the side of the epon block to make serial sectioning of ribbons possible following the protocol of Henry [46] and Ruthensteiner [47]. Bands of ∼20 sections were stained with toluidine blue and mounted in Entellan® (Electron Microscopy Sciences, Pennsylvania, USA). Sections were studied and photographed using light microscopy (BX50 microscope; DP71 camera; Cell^F^ software; Olympus, Japan).

### Benzidine staining of haemoglobin

Using a modified version of the benzidine staining method by Knox [48], haemoglobin was stained in four fixed specimens. A 100% saturated benzidine solution was prepared by adding benzidine to distilled water. The solution was stirred for two hours. Fixed specimens were rinsed in running tap water in the same time period. Specimens were subsequently incubated in the filtered benzidine solution for 1 hour, also under stirring. Next, 3% hydrogen peroxide was added drop by drop until blood vessels turned dark blue. Specimens were either mounted in glycerol directly or dehydrated in a series of alcohol acidified with drops of 0.1% acetic acid, where after tissues were cleared in xylene and mounted in D.P.X between two cover slips. Specimens were analyzed and photographed under a light microscope (BX50 microscope; Dp71 camera; Cell^F^ software; Olympus, Japan).

### Oxygen consumption measurements

O_2_ consumption was measured in seawater kept at ∼100% atmospheric saturation using intermittent respirometry in accordance with Vismann and Hagerman [49]. The experimental setup was placed in a constant temperature room at 6°C. Each experiment encompassed 3–8 measuring sequences consisting of a flushing period of 10–30 min and a measuring period of 30–45 min. The set-up had a chamber flushing rate of 30 ml min^−1^ and a shunt water flow of 8 ml min^−1^ past the O_2_ electrode (E5046, Radiometer Medical ApS, Brønshøj, Denmark).

The O_2_ electrode was connected to a blood/gas monitor (PHM73, Radiometer Medical ApS, Brønshøj, Denmark) and measuring signals were acquired continuously on a PC via data acquisition software (Labtech Notebook Pro version 12.1, Laboratory Technologies Corporation, USA). Prior to each experiment, the O_2_ electrode was calibrated in 0% O_2_ solution (saturated solution of sodium sulphite in borax) and 100% atmospheric O_2_ solution. The O_2_ consumption (MO_2_, µg O_2_ g^−1^ h^−1^) was calculated according to the equation:
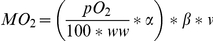
where pO_2_ = the O_2_ partial pressure at full saturation (kPa), ww = wet weight of fixed specimen rinsed for mucus tube (g) (tissue fixed in 4% parafomaldehyde and stored in PBS is assumed to have similar weight as unfixed tissue), α = slope of regression line for O_2_ decrease (% h^−1^), β = O_2_ solubility (µg O_2_ l^−1^ kPa^−1^) and v = volume of the respiration chamber (l).

O_2_ consumption was measured on *O. mucofloris* inhabiting three sectioned cow bone pieces (B1, B2 and B3) and bones in two cuvettes (C1 and C2). Measurements were either initiated directly after the annelids protracted subsequent to the disturbance of being moved (C1, C2, B1) or after one night of acclimatization (B2, B3). All measurements were corrected for background respiration. For B2 and B3 the background respiration was measured using the same water and bones (after dissection and 48 hours of acclimation to restore biological activity). For C1, C2 and B1 the background respiration was the mean value of measurements using new water, bones in two cuvettes and three sectioned bone pieces without *O. mucofloris*. Different respiration chambers were used for sliced bone pieces (volume: 186 ml) and cuvettes (volume: 51 ml).

### Microscale O_2_ measurements

O_2_ concentration profiles were measured in vertical steps of 100 µm with Clark-type O_2_ microelectrodes with a guard cathode [50] (OX-10 Unisense A/S, Aarhus, Denmark) mounted on a manually operated micromanipulator (Märtzhäuser, Wetzlar, Germany) and connected to a picoammeter (PA2000, Unisense A/S, Aarhus, Denmark) and a strip chart recorder (BD25, Kipp&Zonen, Delft, Netherlands). The microelectrodes had a measuring tip diameter of 10 µm, a stirring sensitivity of <1–2% and a 90% response time of <1 second. Linear calibration of the electrode was done from electrode readings in seawater at 100% atmospheric saturation and in anoxic seawater (by addition of sodium dithionite). The O_2_ concentration ([O*_2_*], µmol O*_2_* l^−1^) at each measuring position was calculated according to the equation;
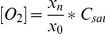
Where x_n_ = O_2_ reading at depth n (pA), x_0_ = O_2_ reading at 100% atmospheric O_2_ saturation (pA) and C_sat_ = O_2_ concentration at 100% atmospheric O_2_ saturation (µmol O_2_ l^−1^). We measured O_2_ concentration profiles from the mixed aerated seawater, across the diffusive boundary layer (DBL) and towards the surface of *O. mucofloris* roots in minke whale bone kept in cuvettes (WB1 and WB2) (Table 2). Prior to these measurements, manually drilled holes (diameter: ∼340 µm) on the side of the plastic cuvettes (Figure 8D, E) were covered with agar (1.5% w/w in seawater) and the cuvettes were left to acclimatize for 2–3 days in order to restore the environment prior to disturbance. The O_2_ concentration was measured through the holes of the cuvette wall towards the enclosed bone surface and further into the bone (Figure 8C). Measurements are given for seven sites on WB1 and five sites on WB2.

The area specific O_2_ flux (J, nmol O_2_ cm^−2^ sec^−1^) at the bone surface was calculated from linear parts of the O_2_ concentration profiles in the DBL and agar plug according to Fick's first law:
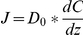
Where dC/dz = the change in O_2_ concentration (dC, nmol O_2_ cm^−3^) with distance (dz, cm), D_0_ = the molecular diffusion coefficient of O_2_ (cm^2^ sec^−1^). We applied a diffusion coefficient of 1.3382 _*_ 10^−5^ cm^2^ sec^−1^, corrected for experimental temperature and salinity.

Additional microscale O_2_ measurements were performed through the mucus tube wall and at the surface of the palps of the individual on WB1. On the mucus tube, the O_2_ concentration was measured from outside the tube and half way into the mucus tube wall. Undisturbed measurements could not be obtained within the centre hole of the tube due to contractions of the worm. On the palps, the O_2_ concentration was measured by keeping the electrode measuring-tip at a fixed position, while the palps approached the tip position as the annelid protracted after being left undisturbed.
